# White Shrimp *Litopenaeus vannamei* That Have Received *Gracilaria tenuistipitata* Extract Show Early Recovery of Immune Parameters after Ammonia Stressing

**DOI:** 10.3390/md13063606

**Published:** 2015-06-05

**Authors:** Yu-Yuan Chen, Jiann-Chu Chen, Yong-Chin Lin, Su-Tuen Yeh, Chien-Lun Huang

**Affiliations:** Department of Aquaculture, College of Life Sciences, National Taiwan Ocean University, Keelung 202, Taiwan; E-Mails: fivedollarboy555@yahoo.com.tw (Y.-Y.C.); d91330003@mail.ntou.edu.tw (Y.-C.L.); d92330006@ntou.edu.tw (S.-T.Y.); m96330013@ntou.edu.tw (C.-L.H.)

**Keywords:** *Litopenaeus vannamei*, *Gracilaria tenuistipitata* extract, ammonia stress, immune parameters, gene expressions

## Abstract

White shrimp *Litopenaeus vannamei* immersed in seawater (35‰) containing *Gracilaria tenuistipitata* extract (GTE) at 0 (control), 400, and 600 mg/L for 3 h were exposed to 5 mg/L ammonia-N (ammonia as nitrogen), and immune parameters including hyaline cells (HCs), granular cells (GCs, including semi-granular cells), total hemocyte count (THC), phenoloxidase (PO) activity, respiratory bursts (RBs), superoxide dismutase (SOD) activity, lysozyme activity, and hemolymph protein level were examined 24~120 h post-stress. The immune parameters of shrimp immersed in 600 mg/L GTE returned to original values earlier, at 96~120 h post-stress, whereas in control shrimp they did not. In another experiment, shrimp were immersed in seawater containing GTE at 0 and 600 mg/L for 3 h and examined for transcript levels of immune-related genes at 24 h post-stress. Transcript levels of lipopolysaccharide and β-1,3-glucan binding protein (LGBP), peroxinectin (PX), cytMnSOD, mtMnSOD, and HSP70 were up-regulated at 24 h post-stress in GTE receiving shrimp. We concluded that white shrimp immersed in seawater containing GTE exhibited a capability for maintaining homeostasis by regulating cellular and humoral immunity against ammonia stress as evidenced by up-regulated gene expression and earlier recovery of immune parameters.

## 1. Introduction

Like other invertebrates, shrimp do not produce immunoglobulin and rely instead on an innate immunity to detect and respond to microbial antigens like lipopolysaccharide (LPS), β-1,3-glucan (βG), and peptidoglycan (PG), known as pathogen-associated molecular patterns (PAMPs) [1,2,3]. PAMP recognition is achieved through pattern-recognition proteins (PRPs) or pattern-recognition receptors (PRRs) that circulate freely in the hemolymph and initiate an immune response that includes phagocytosis, nodule formation, encapsulation, synthesis of antimicrobial peptides, and the propenoloxidase (proPO) cascade [2,4,5]. Lipopolysaccharide and β-1,3-glucan binding protein (LGBP) is an important PRP that occurs in several species of penaeids, including white shrimp *Litopenaeus vannamei* [6,7].

Hemocytes have a crucial role in host immune activity, including cellular and humoral reactions [5]. Among the hemocytes, semi-granular cells (SGCs) and granular cells (GCs) are both induced to degranulate the granules by foreign particles like LPS and βG and subsequently release several proteins including proPO, prophenoloxidase activating enzyme (ppA), peroxinectin (PX), proteinase inhibitors, and lysozyme [4,5]. In the proPO system and proteinase inhibitor system, ppA, proPO, and α2-macroglobulin (α2-M) are important molecules [8,9]. Phenoloxidase (PO), a terminal enzyme in the proPO cascade, is the active form of proPO which is converted by an endogenous trypsin-like serine proteinase, or ppA leading to melanin formation [5]. PO plays a key role in recognition and defense against pathogen infections [10,11]. Peroxinectin (PX) and integrin are the important signaling transduction molecules [8,12,13].

Hyaline cells (HCs) are involved in phagocytosis, an important process for eliminating microorganisms [14,15]. The respiratory burst (RB) that occurs during the process of phagocytosis, leads to the formation of the superoxide anion and other reactive oxygen species (ROS). The superoxide anion and its derivatives are bactericidal [16]. Superoxide dismutase (SOD) scavenges the superoxide anion, prevents the generation of the highly toxic hydroxyl radical (•OH), and catalyses the dismutation of the superoxide anion to form molecular oxygen and hydrogen peroxide [17]. In white shrimp, cytosolic manganese superoxide dismutase (cytMnSOD), mitochondrial manganese superoxide dismutase (mtMnSOD), and extracellular copper-zinc superoxide dismutase (ecCuZnSOD) are important anti-oxidant enzymes ([18,19,20], KP09968). HSP70 is an environmentally inducible heat shock protein (HSP), and acts as a chaperone in regulating normal protein function ([21], AY645906).

The white shrimp is the dominant species currently cultured in the Pacific Rim countries [22]. Shrimp farming has high stocking densities that result in deteriorated environments due to accumulations of organic wastes and metabolic wastes like ammonia, nitrite, and sulfide [23,24]. Ammonia concentration increases directly with the culture period and reaches as high as 7 mg/L ammonia-N (ammonia as nitrogen) in intensive grow-out shrimp farms [23]. The 96-h 50% lethal concentration (median lethal concentration, LC_50_) of ammonia-N on white shrimp (22 ± 2.4 mm length) at 35‰ and 23 °C is 39.54 mg/L [25]. High levels of ammonia in water decrease survival, growth, and osmoregulation of shrimp as well as resistance to *Vibrio alginolyticus*, a pathogenic bacterium isolated from diseased white shrimp [26,27,28]. Therefore, management of optimal water quality parameters including low concentration of ammonia and maintenance of immunity in shrimp are of primary concern.

White shrimp that received red seaweed *Gracilaria tenuistipitata* extract (GTE) via immersion, injection, and diet experienced an enhancement of immune responses [29,30,31]. White shrimp that received GTE via immersion show an earlier recovery of immune parameters after temperature and salinity stressing [32,33]. White shrimp that received GTE via immersion show an earlier recovery of immune parameters after a *Vibrio alginolyticus* challenge and the combined stresses of a *V. alginolyticus* challenge and temperature change [30,33]. White shrimp that received GTE via immersion show lesser decreases in immune parameters after white spot syndrome virus (WSSV) challenges compared to controls [34]. White shrimp fed a diet containing GTE show increased resistance to *V*. *alginolyticus* and WSSV challenges [31]. However, little or nothing is known about immune responses and gene expressions of shrimp that have received the extract under ammonia stressing.

We assume that white shrimp that received GTE may show earlier recovery when subjected to ammonia stress. Accordingly, the purpose of this study was to examine (1) the immune parameters, and (2) transcript levels of LGBP, PX, integrin β (IB), ppA, proPO I, proPO II, α2-M, cytMnSOD, mtMnSOD, ecCuZnSOD, and HSP70 of shrimp immersed in seawater containing GTE and then exposed to ammonia. Immune parameters, HCs, GCs (including semi-granular cells), total hemocyte count (THC), phenoloxidase (PO) activity, RB, superoxide dismutase (SOD) activity, lysozyme activity, and hemolymph protein level were examined.

## 2. Results

### 2.1. Immune Parameters of Shrimp Immersed in Seawater Containing G. tenuistipitata Extract (GTE) Prior to and after Ammonia Stressing

**Figure 1:**
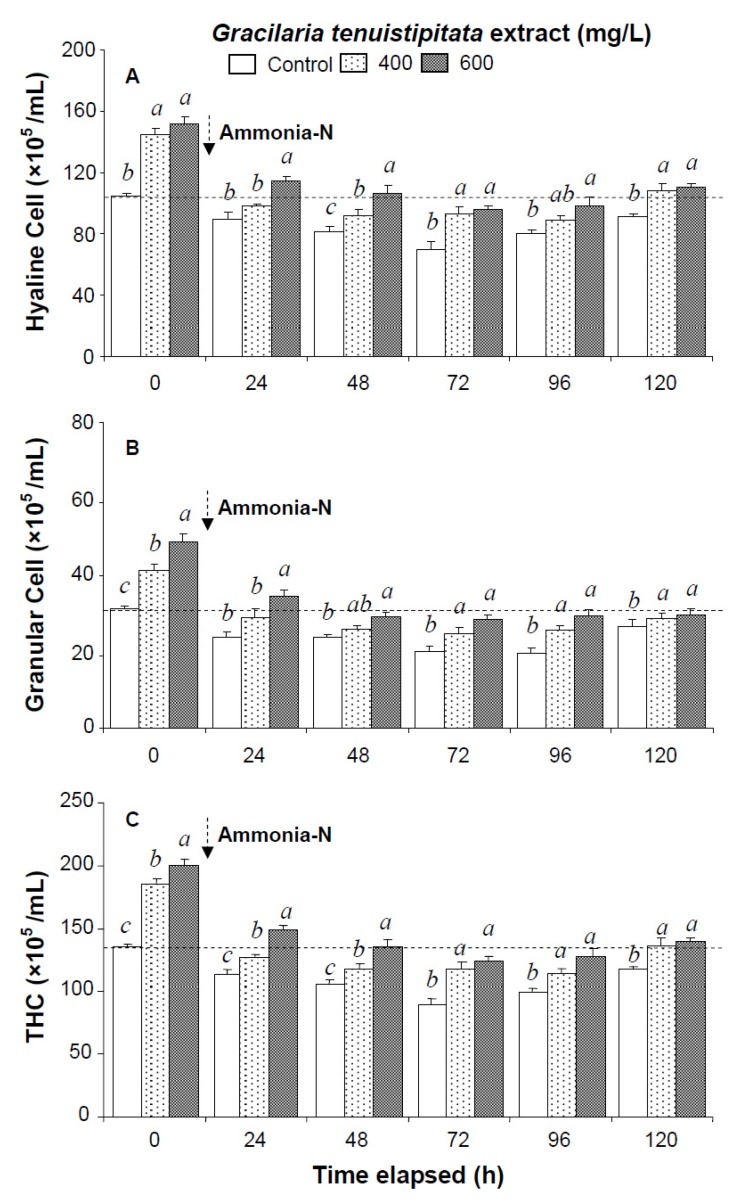
(**A**) Hyaline cells; (**B**) granular cells (including semi-granular cells); and (**C**) total hemocyte count (THC) of white shrimp *Litopenaeus vannamei* immersed in seawater containing *Gracilaria tenuistipitata* extract at 0 (control), 400, and 600 mg/L for 3 h and then exposed to 5 mg/L ammonia-N for 24, 48, 72, 96, and 120 h. Each bar represents the mean value from eight determinations with the standard error (SE). Data (mean ± SE) with different letters are significantly different (*p* < 0.05) among treatments.

**Figure 2:**
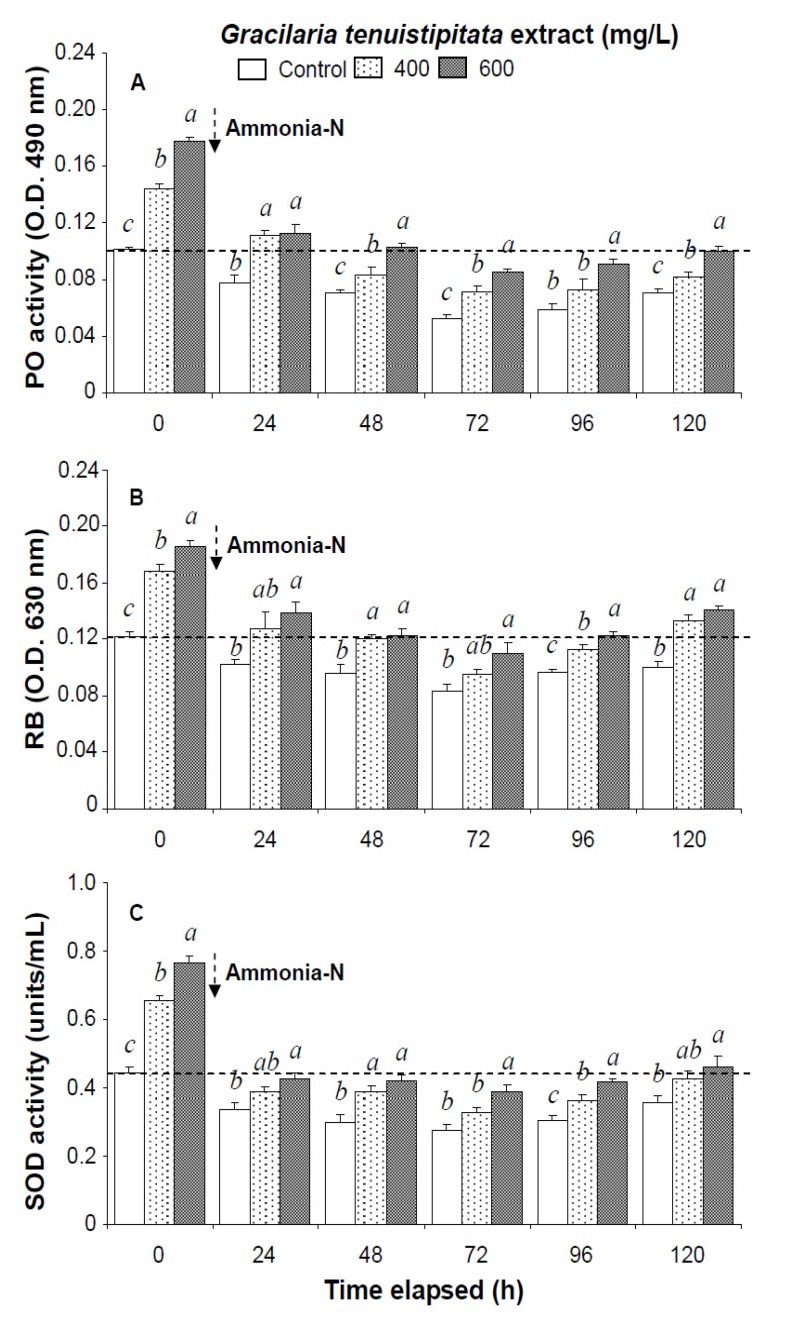
(**A**) Phenoloxidase activity; (**B**) respiratory burst (RB); and (**C**) superoxide dismutase (SOD) activity of white shrimp *Litopenaeus vannamei* immersed in seawater containing *Gracilaria tenuistipitata* extract at 0 (control), 400, and 600 mg/L for 3 h and then exposed to 5 mg/L ammonia-N for 24, 48, 72, 96, and 120 h. Data (mean ± SE) with different letters are significantly different (*p* < 0.05) among treatments

**Figure 3:**
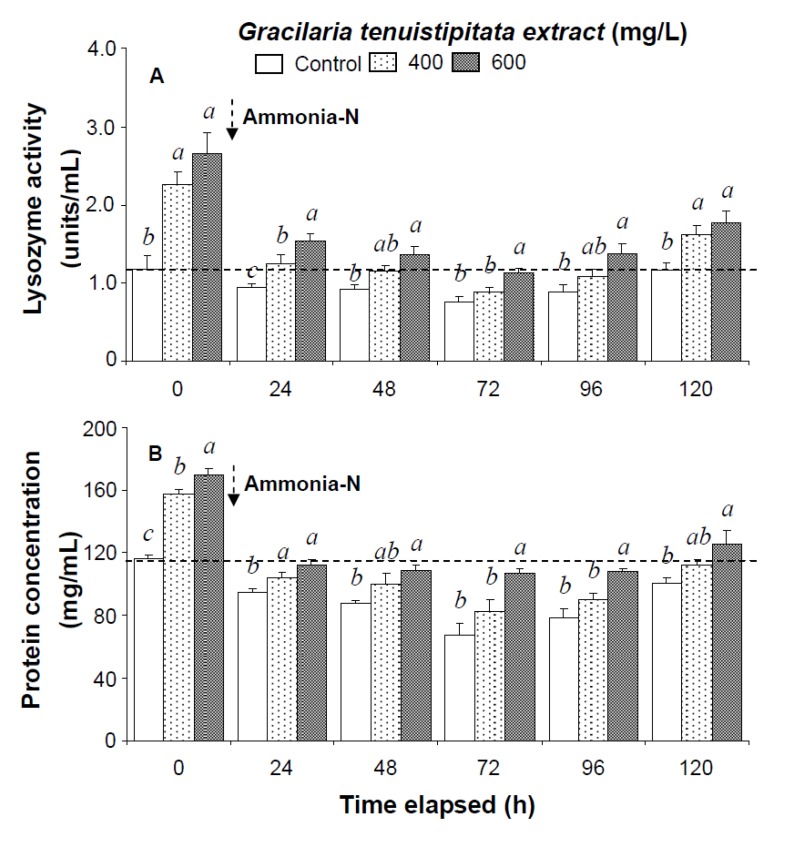
(**A**) Lysozyme activity and (**B**) hemolymph protein level of white shrimp *Litopenaeus vannamei* immersed in seawater containing *Gracilaria tenuistipitata* extract at 0 (control), 400, and 600 mg/L for 3 h and then exposed to 5 mg/L ammonia-N for 24, 48, 72, 96, and 120 h. Data (mean ± SE) with different letters are significantly different (*p* < 0.05) among treatments.

Prior to the ammonia stress test, immune parameters increased directly with concentration of GTE. The levels of HC, GC, and THC of shrimp immersed in seawater containing 400 mg/L GTE increased by 38%, 33%, and 37%, respectively. PO activity, RB, SOD activity, lysozyme activity, and hemolymph protein level of shrimp immersed in 400 mg/L GTE increased significantly by 43%, 39%, 47%, 79%, and 36%, respectively (Figure 1, Figure 2 and Figure 3).

All shrimp in the three ammonia test treatments were still alive after 120 h. Hemocyte counts and other immune parameters from the three treatments decreased with exposure time after 24–72 h but increased after 96 h and 120 h. HC, GC, and THC from shrimp immersed in 600 mg/L GTE returned to their original values after 120 h. However, HC, GC, and THC from control shrimp did not return to background values after 120 h (Figure 1).

In the ammonia stress test, PO activity, RB, and SOD activity of shrimp immersed in seawater containing 600 mg/L GTE returned to their original values by 120 h, 96 h, and 120 h, respectively. RB and SOD activity of shrimp immersed in 400 mg/L GTE both returned to their original values by 120 h. However, PO activity, RB, and SOD activity of control shrimp did not return to their original values after 120 h (Figure 2). In the ammonia stress test, lysozyme activity and hemolymph protein levels of shrimp immersed in 600 mg/L GTE returned to their original values at 96 h and 120 h, respectively. However, lysozyme activity and hemolymph protein levels of control shrimp did not return to original values after 120 h (Figure 3).

### 2.2. Transcript Levels of LGBP, PX, IB, ppA, proPO I, proPO II, α2-M, cytMnSOD, mtMnSOD, ecCuZnSOD, and HSP70 in Shrimp Immersed in Seawater Containing G. tenuistipitata Extract (GTE) Prior to and after Ammonia Stressing

**Figure 4:**
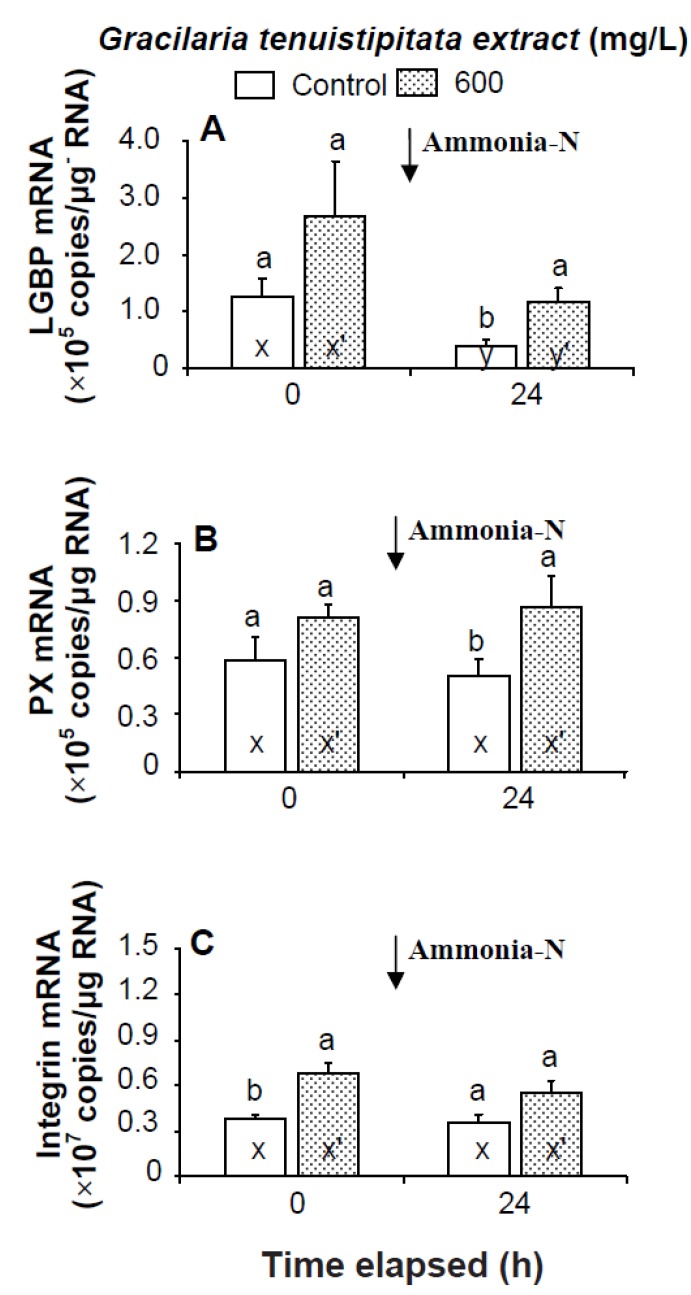
Transcript levels of (**A**) lipopolysaccharide and β-1,3-glucan binding protein (LGBP); (**B**) peroxinectin (PX); and (**C**) integrin β (IB) of hemocytes of white shrimp *Litopenaeus vannamei* immersed in seawater containing *Gracilaria tenuistipitata* extract (GTE) at 0 (control) and 600 mg/L for 3 h and then exposed to seawater containing 5 mg/L ammonia-N for 24 h. Each bar represents the mean value from eight determinations with the standard error (SE). Data (mean ± SE) with different letters (a, b) are significantly different (*p* < 0.05) between treatments. Data with different letters (x, y) and with different letters (x′, y′) are significantly different *(p* < 0.05) before and after ammonia stress between control shrimp and between GTE receiving shrimp.

**Figure 5:**
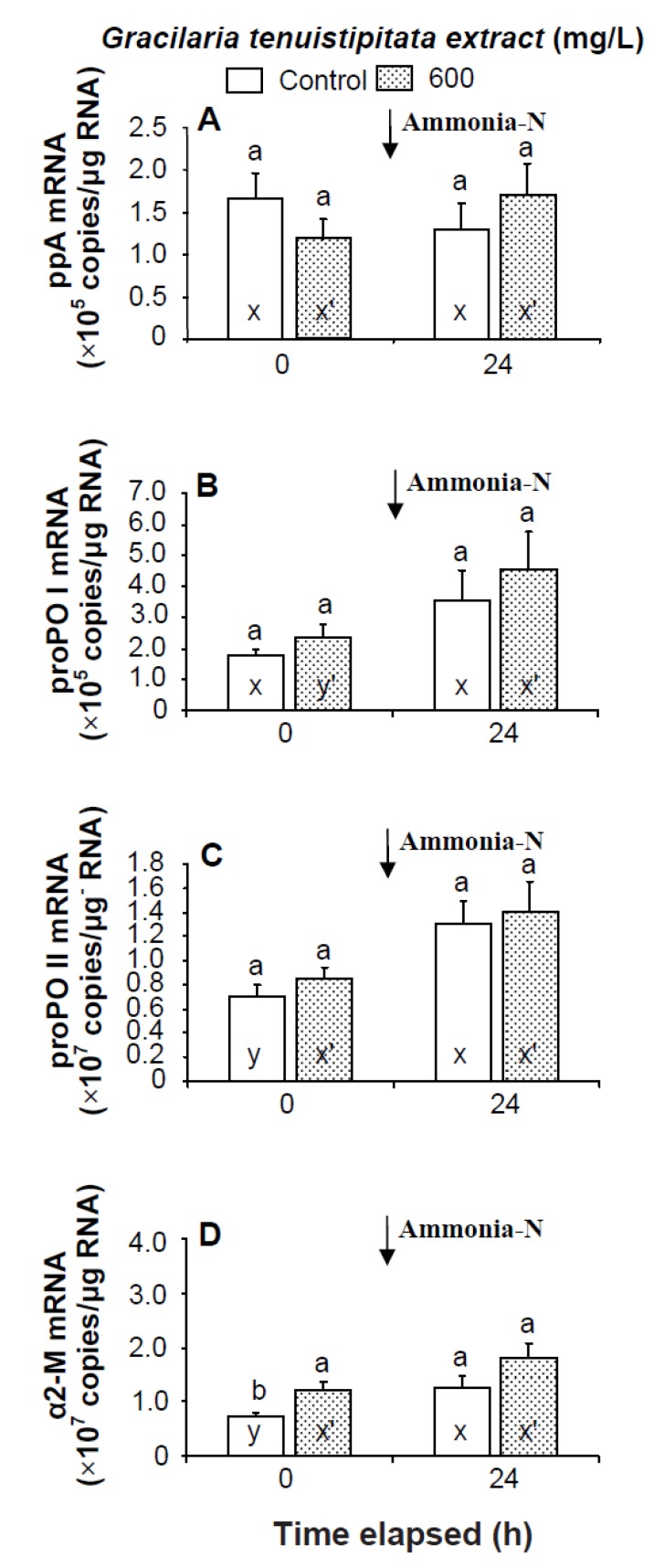
Transcript levels of (**A**) prophenoloxidase activating enzyme (ppA); (**B**) prophenoloxidase I (proPO I); (**C**) proPO II; and (**D**) α2-macroglobulin (α2-M) of hemocytes of white shrimp *Litopenaeus vannamei* immersed in seawater containing *Gracilaria tenuistipitata* extract at 0 (control) and 600 mg/L for 3 h and then exposed to seawater containing 5 mg/L ammonia-N for 24 h. Data with different letters (x, y) and with different letters (x′, y′) are significantly different *(p* < 0.05) before and after ammonia stress between control shrimp and between GTE receiving shrimp.

Prior to the ammonia stress test, IB and α2-M transcript levels of shrimp immersed in seawater containing 600 mg/L GTE were significantly up-regulated (*p* < 0.05). Transcript levels of LGBP, PX, proPOI, proPO II, and α2-M of shrimp immersed in 600 mg/L GTE increased but were not significantly different (*p* > 0.05) from control shrimp (Figure 4 and Figure 5). In the ammonia stress test, LGBP and PX transcript levels of shrimp immersed in 600 mg/L GTE were significantly (*p* < 0.05) higher than in control shrimp after 24 h. The transcript levels of IB, ppA, proPO I, proPO II, and α2-M of shrimp immersed in 600 mg/L GTE increased but were not significantly different (*p* > 0.05) from control shrimp after 24 h of post-ammonia stressing (Figure 4 and Figure 5).

**Figure 6:**
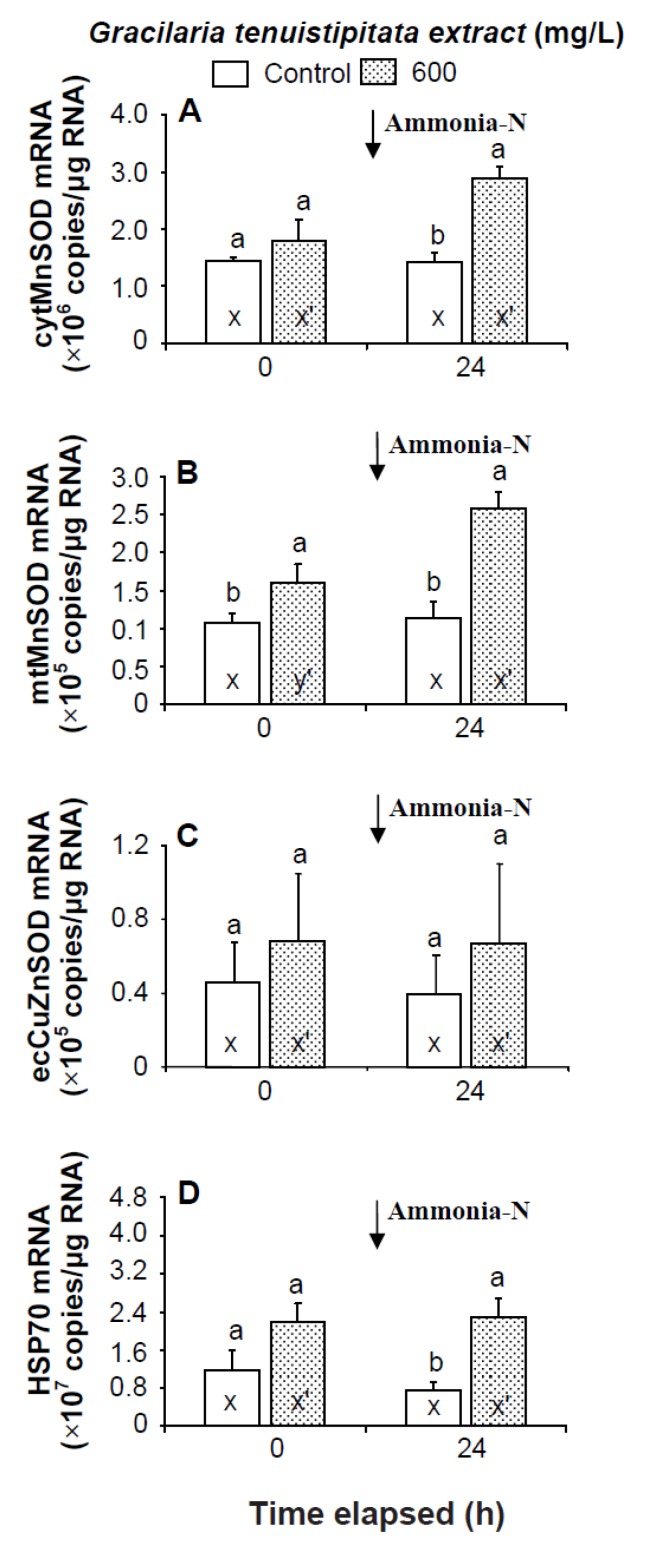
Transcript levels of (**A**) cytosolic manganese superoxide dismutase (cytMnSOD), (**B**) mitochondrial manganese superoxide dismutase (mtMnSOD), (**C**) extracellular copper-zinc superoxide dismutase (ecCuZnSOD), and (**D**) heat shock protein 70 (HSP70) of hemocytes of white shrimp *Litopenaeus vannamei* immersed in seawater containing *Gracilaria tenuistipitata* extract at 0 (control) and 600 mg/L for 3 h and then exposed to seawater containing 5 mg/L ammonia-N for 24 h. Data with different letters (x, y) and with different letters (x′, y′) are significantly different *(p* < 0.05) before and after ammonia stress between control shrimp and between GTE receiving shrimp.

Prior to the ammonia stress test, the transcript level of mtMnSOD of shrimp immersed in seawater containing 600 mg/L GTE was significantly up-regulated (*p* < 0.05). The transcript levels of cytMnSOD, ecCuZnSOD, and HSP70 of shrimp immersed in 600 mg/L GTE were up-regulated but not significantly different (*p* > 0.05) from control shrimp. In the ammonia stress test, the transcript levels of cytMnSOD, mtMnSOD, and HSP70 were significantly higher (*p* < 0.05) in shrimp immersed in 600 mg/L GTE than in control shrimp after 24 h. The transcript level of ecCuZnSOD of shrimp immersed in 600 mg/L GTE was up-regulated but not significantly different (*p* > 0.05) from control shrimp following ammonia stressing (Figure 6).

## 3. Discussion

The mortality of white shrimp *L. vannamei* subjected to combined stressing from a *V. alginolytcius* challenge and ammonia exposure is much higher than in shrimp subjected to ammonia alone [28]. White shrimp exposed to ammonia and then receiving a *V. alginolyticus* challenge show decreased phagocytic activity and clearance efficiency compared to controls [28]. Phagocytic and antibacterial activity in the swimming crab *Portunus trituberculatus* exposed to 1 and 5 mg/L ammonia-N decreased after 6 h [35]. Therefore, shrimp and crabs show decreased resistance against pathogens in the presence of ammonia in water.

In the present study, the immune parameters including hemocyte counts, PO activity, RB, and SOD activity significantly increased in shrimp that immersed in seawater containing GTE after 3 h. Several scientists indicated that shrimp hemocytes incubated with βG, fucoidan, and carrageenan exhibit degranulation of granules, changes in cell size and viability, and increased PO activity and RB [36,37,38]. White shrimp that received carrageenan via immersion showed increased hemocyte count, and higher number of mitotic cells in the hematopoietic tissue (HPT) [36]. We conjecture that GTE like its analogue, carrageenan not only cause degranulate hemocytes but also induces hemocyte death that results in hematopoiesis.

The immune parameters decreased in shrimp following environmental stressing. For instance, the hemocyte count of blue shrimp *Litopenaeus stylirostris* exposed to 1.5 and 3.0 mg/L ammonia-N decreased by 15% and 50%, respectively after 12 h [39]. The THC of the swimming crab exposed to 1 mg/L ammonia-N decreased after 6 h [35]. The PO activity of white shrimp exposed to 5.24 mg/L ammonia-N decreased significantly (*p* < 0.05) after 168 h [28]. In the present study, immune parameters (hemocyte count, PO activity, RB, SOD activity, and lysozyme activity) of control shrimp exposed to 5 mg/L ammonia-N significantly decreased (*p* < 0.05) earlier (after 24 h), whereas the immune parameters of shrimp that immersed in seawater containing GTE at 600 mg/L decreased later (after 72 h) at post ammonia stress. Therefore, shrimp that received GTE showed late decrease and earlier recovery in immune parameters compared to their original values indicating a protective immune response against ammonia stressing.

The immune parameters of shrimp that immersed in seawater containing GTE and then subjected to salinity stress (from 35% to 25%) returned to their original values earlier than did those of control shrimp that subjected to salinity stressing [32]. The immune parameters of shrimp that immersed in seawater containing GTE and then subjected to temperature stressing (from 24 °C to 28 °C) returned to their original values much earlier than did those of control shrimp that subjected to temperature stress [33]. The immune parameters of shrimp that fed a diet containing GTE at 0.5 g/kg, and then subjected to ammonia stressing returned to their original values earlier than did those of control shrimp and then subjected to ammonia stressing [40]. In the present study, the immune parameters of shrimp that received 600 mg/L GTE via immersion returned to their original values earlier than did those of control shrimp after ammonia stressing. We suggest that shrimp immersed in seawater containing the GTE show protective immunity or an immunity buffering effect on proPO activation system and phagocytosis system [4]. Shrimp that received GTE showed a capability for positive regulation by an earlier recovery of immune parameters in maintaining homeostasis.

Antioxidant enzymes are known to play an important role in shrimp defense by scavenging the superoxide anion and other ROS [41]. In the present study, SOD activity in shrimp that received GTE was significantly higher than in control shrimp prior to ammonia stressing and at 24~120 h post-stress. The mtMnSOD transcript level of shrimp that received GTE was up-regulated. The cytMnSOD and mtMnSOD transcript levels of shrimp that received GTE were up-regulated at 24 h post-stress. Therefore, shrimp that received GTE showed an anti-oxidant capability against oxidative stress induced by ammonia.

Transcript levels of immune-related genes are affected in shrimp injected with foreign particles. For instance, the LGBP transcript in the hemocytes of kuruma shrimp *Marsupenaeus japonicus* is up-regulated within 12~72 h after being injected with LPS [7]. The LGBP transcript in the hemocytes of white shrimp is up-regulated within 12 h after being injected with *V. alginolyticus* [6]. The PX transcript in the hemocytes of white shrimp is up-regulated within 6~24 h after being injected with *V. alginolyticus* [42]. The LGBP transcript of white shrimp fed a diet containing βG is up-regulated within 72 h post-feeding [43,44]. The transcripts of LGBP, IB, PX, ppA, propO I, proPO II, and α2-M are up-regulated in shrimp receiving a diet containing carrageenan at 0.5 g/kg after 3 weeks [36]. LGBP contains two potential polysaccharide recognition motifs (polysaccharide binding motif and β-glucan recognition motif) and is known to bind to microbes and transmit signals to hemocytes through cell surface receptors [5,7]. Recent evidence strongly implies that recombinant LGBP binds with LPS and β-glucan, and subsequently leading to an increase PO activity in tiger shrimp *P. monodon* [44]. In a preliminary study, we also found that LGBP can bind with GTE and that PO activity increased in shrimp hemocytes treated with LGBP-GTE mixture [45].

The transcript levels of immune-related shrimp genes are affected by environmental stresses. For instance, the PX transcript of white shrimp subjected to higher temperature stress (raised to 32 °C from 26 °C) was reduced after two days [12]. The LGBP and α2-M transcripts of white shrimp subjected to salinity stress (lowered to 25‰ from 35‰) were up-regulated after 24 and 12 h, respectively [32]. The LGBP, PX and IB transcripts of white shrimp subjected to pH stress (lowered to pH 6.8 from pH 8.2) are down-regulated after 24 h [46]. The transcript of HSP70, a stress-related gene, is up-regulated in tiger shrimp *P. monodon* after heat shock treatment (raised from 26 °C to 37 °C) [47]. Crustin, anti-lipopolysaccharide factor (ALF), and lysozyme expressions are down-regulated in the swimming crab after exposure to 20 mg/L ammonia-N, whereas α2-M expression is up-regulated after 6 h [35]. In the present study, LGBP expression was down-regulated, whereas proPO II and α2-M expressions were up-regulated in control shrimp exposed to ammonia. We suggest that exposing white shrimp to ammonia may decrease the expressions of antimicrobial peptides like ALF, crustin, lysozyme, and penaeidin.

LGBP, PX, and α2-M transcripts of shrimp immersed in seawater at 35‰ salinity containing 600 mg/L GTE were up-regulated within 12 h after stressing with 25‰ salinity [32]. LGBP, PX, and IB transcripts of shrimp immersed in seawater (35‰; pH 8.2) containing *S. platensis* extract at 600 mg/L were up-regulated within 24 h, 6 h, and 24 h, respectively, post-stress at pH 6.8 [46]. PX has cell adhesion and is associated with IB in mediating the binding of hemocytes, PAMP, and PRP [4]. In the present study, shrimp receiving GTE showed increases in the expressions of LGB, PX, and HSP70 after ammonia stressing. This suggests that the LGBP-GTE complex may transfer a signal to hemocytes and enhance LGBP synthesis in shrimp receiving GTE that are then subjected to ammonia stressing. Further research is needed to identify the recognition and binding of LGBP with GTE or its analogue like carrageenan in shrimp prior to and after ammonia stressing.

In conclusion, white shrimp *L. vannamei* immersed in seawater containing *G. lenuistipitata* extract that contains galactose-polymer as main effective molecules were capable of maintaining immune homeostasis as evidenced by earlier recovery in immune parameters and up-regulated transcript levels of LGBP, PX, cytMnSOD, mtMnSOD, and HSP70 while under ammonia stressing.

## 4. Materials and Methods

### 4.1. Preparation of G. tenuistipitata Extract (GTE)

*Gracilaria tenuistipitata* was collected from an algae farm in southern Taiwan. The preparation of the extract was followed the previously described procedures [31]. The GTE contains 11.72% crude protein, 0.49% crude lipid, 42.75% ash, and 45.04% carbohydrate (nitrogen free extract and crude fiber). GTE galacton content as quantified for galactose by the phenol-sulfuric acid method and sulfate content as measured by the gelatin-barium chloride turbidimetric method are 50.7% and 11%, respectively [48,49].The main polysaccharide components are galactose (60.0%), fucose (35.0%), fructose (3.3%), and glucose (1.8%) based on gas chromatographic-mass spectroscopic (GC-MS) analysis after hydrolytic reduction and acetylation of sugars [50,51].

### 4.2. Experimental Design for the Immersion Test

White shrimp *L. vannamei* obtained from the University Animal Center were shipped to the laboratory, and acclimated in fiberglass tanks having sand bed air lifts providing constantly aerated water and quarantined for two weeks prior to the experiments. Average shrimp weight was 11.2 ± 0.6 g (mean ± SD) with no significant size differences among treatments. Only shrimp in the intermoult stage were used in this study [52]. Two studies were conducted, the first to examine immune parameters and transcript levels of immune-related genes after shrimp were immersed in seawater containing GTE and the second to examine immune parameters and transcript levels of immune-related genes after shrimp were immersed in seawater containing GTE and then ammonia stressed. The first study tested the shrimp prior to ammonia stressing and the second tested them after ammonia stressing. Water temperatures during experimental periods ranged 27–28 °C, pH 7.9–8.1, salinity 35‰, and dissolved oxygen (DO) 5.63–6.72 mg/L. In addition, shrimp that received no GTE and no ammonia exposure served as control.

### 4.3. Immune Parameters of Shrimp Immersed in Seawater Containing G. tenuistipitata Extract (GTE) Prior to and after Ammonia Stressing

White shrimp that had been immersed in seawater containing GTE for 3 h were then exposed to 5 mg/L ammonia-N. There were three GTE solutions (0, 400, and 600 mg/L) with one exposure time (3 h) prior to stress, and three solutions of GTE (0, 400, and 600 mg/L) with five exposure times (24, 48, 72, 96, and 120 h) in the ammonia stress test. Prior to the ammonia stress test, 10 shrimp that had been immersed in each test solution (20 L) for 3 h were sampled. Therefore, there were three treatments, and 30 shrimp (10 × 3) in total were used for the study prior to the ammonia stress test. In the ammonia stress test, 50 shrimp immersed in each test solution for 3 h were sampled and then exposed to 5 mg/L ammonia-N. Ten shrimp were used in each test solution and each exposure time. Therefore, there were 15 treatments, and 150 shrimp (10 × 3 × 5) in total were used for the ammonia stress test. The concentration of ammonia-N test solution (5 mg/L) was determined based on one eighth of the 96-h median lethal concentration (LC_50_) (39.54 mg/L) of ammonia-N on white shrimp juveniles at 35‰ and 23 °C [25]. The test water solution (5 mg/L) was renewed daily.

### 4.4. Measurements of Immune Parameters

At 3 h prior to the ammonia stress test, and after 24, 48, 72, 96, and 120 h in the ammonia stress test, eight shrimp from each tank were sampled individually. Sampling of hemolymph, preparation of anticoagulant-hemolymph mixture, and hemocyte counts followed the procedures previously described [32]. Briefly, hemolymph (300 µL) was individually withdrawn from the ventral sinus of each shrimp using a 1-mL sterile syringe with a 25 gauge needle, diluted with 2700 µL of an anticoagulant solution (30 mM trisodium citrate, 340 mM sodium chloride, and 10 mM EDTA at pH 7.55, with osmolality adjusted to 718 mOsm/kg by the addition of 115 mM glucose). The hemolymph-anticoagulant mixture was placed in four tubes. Each tube contained 500, 1000, 1000, and 500 µL of the hemolymph mixture, and was used to measure (1) hemocyte count, RB, and hemolymph protein concentration; (2) PO activity; (3) SOD activity; and (4) lysozyme activity, respectively [32]. Hyaline cells and granular cells can be identified based on their size and degree of granularity [2].

PO activity was measured spectrophotometrically by recording the formation of dopachrome produced from l-dihydroxyphenylalanine (l-DOPA) [30,53,54]. Hemocyte RB was quantified by measuring the intracellular production of the superoxide anion using the reduction of nitroblue tetrazolium (NBT) to formazan [30,55]. SOD activity was measured by its ability to inhibit superoxide radical-dependent reactions using a Ransod Kit (Randox, Crumlin, UK) [32,56]. Lysozyme activity was determined following a previously described method [57,58,59]. Hemolymph protein level was quantified with a Bio-Rad protein assay kit no. 500-0006 (Bio-Rad Laboratories, Richmond, CA, USA) with bovine serum albumin (molecular weight: 66 kDa) as a standard [60,61].

### 4.5. Transcript Levels of LGBP, PX, IB, ppA, proPO I, proPO II, α2-M, cytMnSOD, mtMnSOD, ecCuZnSOD, and HSP70 in Shrimp Immersed in Seawater Containing G. tenuistipitata Extract (GTE) Prior to and after Ammonia Stressing

There were two solutions of GTE (0 and 600 mg/L) with one exposure time (3 h) prior to the ammonia stress and two GTE solutions (0 and 600 mg/L) with one exposure time (24 h) after the ammonia stress. Eight shrimp from each concentration and exposure time were sampled for gene expression assays. There were a total of 4 treatments and 32 shrimp [(8 × 2 × 1) + (8 × 2 × 1)] used in the study.

Hemolymph (500 µL) was individually withdrawn as described above, placed in a tube containing 500 µL of an anticoagulant solution, and centrifuged at 800× *g* and 4 °C for 20 min. The isolation of total RNA from hemocytes, design of primer sets for each gene expression, and quantification of target genes LGBP, PX, IB, ppA, proPO I, proPO II, α2-M, cytMnSOD, mtMnSOD, ecCuZnSOD, and HSP70, and the internal control (EF1α) were measured with qPCR [62]. Primer sets for each gene were designed based on the available genes of white shrimp using Beacon Designer Software vers. 6.0 (Table 1).

**Table 1:** Primers used for the quantitative real-time PCR study of elongation factor 1-alpha (EF 1α) and eleven immune-related genes of the white shrimp *Litopenaeus vannamei*.

Gene	Primer Name	Sequence 5′ to 3′	Amplicon	Reference/GenBank
*LGBP*	Liva LGBP qPCR F	CGG CAA CCA GTA CGG AGG AAC	115 bp	EU102286
	Liva LGBP qPCR R	GTG GAA ATC ATC GGC GAA GGA G		
*Peroxinectin*	Liva PX qPCR F	ATC CAG CAG CCA GGT ATG	147 bp	[12]
	Liva PX qPCR R	CAG ACT CAT CAG ATC CAT TCC		
*Integrin β*	Liva It β qPCR F	TTG GGC ATC GTG TTC GGA CTC	184 bp	GQ889365
	Liva It β qPCR R	TGA AGG TGT TGG TCG CAG GTC		
*ppA*	Liva ppA qPCR F	CTA GAG ACG TCG GTG TCA TCA CC	151 bp	AY368151
	Liva ppA qPCR R	AAC TTG CCG TCC GAA GTG CG		
*proPO I*	Liva proPO I qPCR F	ACG TCA CTT CCG GCA AGC GA	156 bp	AY723296
	Liva proPO I qPCR R	CCT CCT TGT GAG CGT TGT CAG G		
*proPO II*	Liva proPO II qPCR F	ACC ACT GGC ACT GGC ACC TCG TCT A	161 bp	EU373096
	Liva proPO II qPCR R	TCG CCA GTT CTC GAG CTT CTG CAC		
*α2-macroglobulin*	Liva A2M qPCR F	GCA CGT AAT CAA GAT CCG	204 bp	DQ988330
	Liva A2M qPCR R	CCC ATC TCA TTA GCA CAA AC		
*cytMnSOD*	Liva cytMnSOD qPCR F	TGA CGA GAG CTT TGG ATC ATT CC	155 bp	DQ029053
	Liva cytMnSOD qPCR R	TGA TTT GCA AGG GAT CCT GGT T		
*mtMnSOD*	Liva mtMnSOD qPCR F	CAG ACT TGC CCT ACG ATT AC	216 bp	KP099968
	Liva mtMnSOD qPCR R	AGA TGG TGT GAT TGA TGT GAC		
*ecCuZnSOD*	Liva CuZnSOD qPCR F	CGC GGG AGA CAC AGC TGA TTT C	164 bp	HM371157
	Liva CuZnSOD qPCR R	GAA ATC CAG GGT GCC GGA GA		
*HSP70*	Liva Hsp70 qPCR F	CCT CCT ACG TCG CCT TCA CAG ACA	233 bp	AY645906
	Liva Hsp70 qPCR R	GGG GTA GAA GGT CTT CTT GTC TCC C		
EF1α	Liva EF1α qPCR F	ATG GTT GTC AAC TTT GCC CC	500 bp	GU136229
	Liva EF1α qPCR R	TTG ACC TCC TTG ATC ACA CC		

### 4.6. Statistical Analysis

All data were subjected to a one-way analysis of variance (ANOVA). If significant differences were indicated at the 0.05 level, then Tukey’s multiple-comparisons test was conducted to examine significant differences among treatments using SAS computer software (SAS Institute, Cary, NC, USA). Statistical significance of differences required that *p* < 0.05.
